# Optimization of Temperature Modulation for Gas Classification Based on Bayesian Optimization

**DOI:** 10.3390/s24092941

**Published:** 2024-05-06

**Authors:** Tatsuya Iwata, Yuki Okura, Maaki Saeki, Takefumi Yoshikawa

**Affiliations:** 1 Department of Electrical and Electronic Engineering, Toyama Prefectural University, Imizu 939-0398, Japantyoshikawa@pu-toyama.ac.jp (T.Y.); 2 Department of Information Systems Engineering, Toyama Prefectural University, Imizu 939-0398, Japan; yuki-okura@pu-toyama.ac.jp

**Keywords:** gas sensors, temperature modulation, electronic nose, gas classification, Bayesian optimization

## Abstract

This study proposes an optimization method for temperature modulation in chemiresistor-type gas sensors based on Bayesian optimization (BO), and its applicability was investigated. As voltage for a sensor heater, our previously proposed waveform was employed, and the parameters determining the voltage range were optimized. Employing the Bouldin–Davies index (DBI) as an objective function (OBJ), BO was utilized to minimize the DBI calculated from a feature matrix built from the collected data followed by pre-processing. The sensor responses were measured using five test gases with five concentrations, amounting to 2500 data points per parameter set. After seven trials with four initial parameter sets (ten parameter sets were tested in total), the DBI was successfully reduced from 2.1 to 1.5. The classification accuracy for the test gases based on the support vector machine tends to increase with decreasing the DBI, indicating that the DBI acts as a good OBJ. Additionally, the accuracy itself increased from 85.4% to 93.2% through optimization. The deviation from the tendency that the accuracy increases with decreasing the DBI for some parameter sets was also discussed. Consequently, it was demonstrated that the proposed optimization method based on BO is promising for temperature modulation.

## 1. Introduction

The information on smells has the potential to be utilized in various fields including quality assessment in the food industry, environmental monitoring, and healthcare [1,2,3]. An electronic nose (e-nose), generally composed of a sensor array, a signal processing unit, and a pattern recognition unit, has been applied to these fields, e.g., the classification of food quality/origin [4,5,6,7,8], hazardous gas detection [9], and breath analysis [10,11,12]. Due to its features of a compact and low-cost system compared with conventional analysis systems [9,13], e-nose can open up new applications like fast screening in the industry and daily use at home for such fields as listed above.

For this purpose, an e-nose is required to achieve high classification accuracy. Classification is usually carried out on feature vectors extracted from the responses followed by pattern recognition. Steady-state responses from the elements of a sensor array are the most straightforward and common way to build a feature vector. In contrast, transient responses, originating from the dynamics of the physical/chemical interaction between gas molecules and a sensor material, are favorable in terms of containing helpful information about smells compared to those in the steady state. As transients, responses at the onset of exposure to a target smell [14,15], the onset of ventilation [16], and during the changes in sensor temperature [17] are most frequently employed. Among these, intentional sensor temperature changes known as temperature modulation in chemiresistor sensors are most frequently used. The temperature is the most critical parameter in the interaction between a gas and a sensor material. Additionally, it is easily controlled by an electrical signal (e.g., voltage) applied to the heater, which allows for choosing an appropriate temperature profile. Based on the temperature modulation, a feature vector is constructed from the time-dependent response of sensors, which allows gas classification using a single sensor. Needless to say, temperature modulation is also applicable to a sensor array, for which it is seen as a way to increase the data dimension [18]. As the voltage waveform for the temperature modulation, periodical wave (sinusoidal, rectangular, etc.) [19,20,21,22,23], stepwise [24,25,26], pulse width modulation [27], and multi-sinusoidal waves [28,29] were employed, and their applicability has been proven thorough gas classification. Experimentally, it has been shown that the amplitude and frequency significantly affect the gas selectivity and that the hysteresis effects also include plentiful information for gas classification [22,30,31,32]. The authors proposed the waveform whose amplitude and frequency periodically change and demonstrated its promising properties in fast data acquisition and precise gas classification [33,34].

On the other hand, such high flexibility for the temperature profile makes its appropriate choice for classification a standing problem. For gas classification, collecting a calibration dataset is necessary to examine the goodness of a parameter set, which is a time-consuming process. Thus, it is difficult to carry out exhaustive tests for all the possible parameter sets to find the most appropriate one. To date, several approaches have been proposed to determine systematically the suitable temperature profile. Vergara et al. employed multi-level pseudo-random sequences as heater voltage to estimate the impulse response of the sensor [28,29]. The so-called resolution power was calculated on the spectral components of the impulse response, from which optimal frequencies to form a multi-frequency sinusoidal wave were selected. Approaches that inversely control the temperature using the feedback from the sensor output have also been proposed. Martinelli et al. employed pulses as the heater voltage, where the pulse width was determined adaptively by a feedback signal from the sensor resistance [35,36]. Under a steady state, the widths of a train of pulses, which varies with time, were used as a feature vector. Herrero-Carrón et al. adopted the temperature profile as a feature vector, where a PID-based temperature control was introduced to bring the sensor output close to a reference value [37]. Gosangi and Gutierrez-Osuna proposed active temperature modulation where the operating temperature was actively modulated so that the belief, which assigned a probability to a gas concentration profile, was maximized based on a probabilistic basis [38,39]. Although it has been shown that these techniques are successful, they are only applicable to specific waveforms and/or modulation schemes. A general method to determine the temperature profile has not yet been established, and in many cases, the optimization was still done empirically with the consideration of, e.g., the responses to target gases under static measurements [21,23,25,26]. However, it is impractical to empirically search an appropriate parameter set for complex waveforms with a lot of parameters, like the one the authors proposed, which has nearly ten parameters as shown later.

Then, the authors propose to employ Bayesian optimization (BO) to determine an appropriate parameter set in the temperature modulation with a reduced number of examinations of parameter sets. BO is an optimization method based on Gaussian process regression (GPR) and applied in many fields, including machine learning (ML) [40] and materials informatics [41]. In particular, BO is successful in materials synthesis, which is a time-consuming process, and the number of synthesis trials is practically limited [42,43]. In this study, BO was applied to determine the appropriate parameter set for the temperature modulation. Through the choice of the objective function and evaluation of classification accuracy by the ML model, the applicability of BO to the parameter optimization for the temperature modulation is investigated.

## 2. Experimental Methods

### 2.1. Heater Waveform and Optimized Parameters

**Figure 1:**
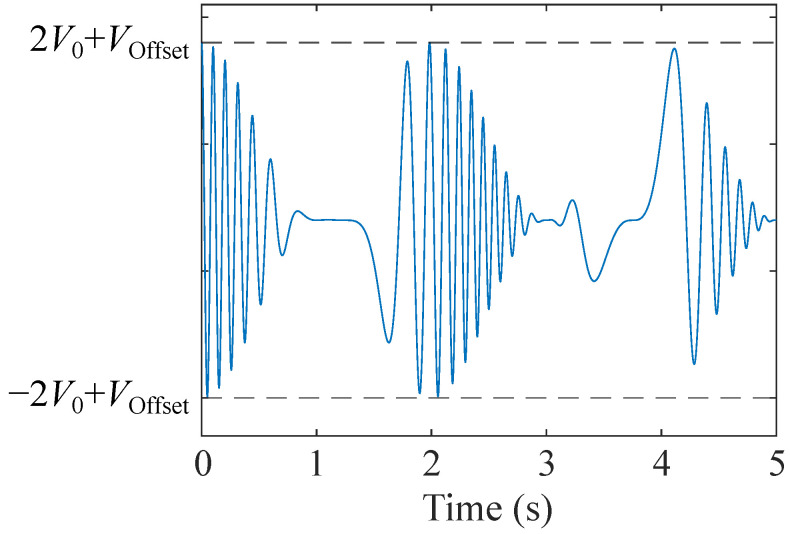
$V_{H}$ waveform employed in this study. $V_{0}$ and $V_{\mathrm{Offset}}$ determine the peak and bottom of the wave, and hence, the range of $V_{H}$.

The heater voltage ($V_{H}$) used in this study is expressed by the following equation [33]:

(1) $$V_{H}\left(t\right)=V_{0}\left\{1+\cos\left(2\pi f_{1}t\right)+\phi_{1}\right\}\cos\left\{2\pi f_{0}t+\frac{\Delta f}{f_{2}}\sin\left(2\pi f_{2}t+\phi_{2}\right)+\phi_{3}\right\}+V_{\mathrm{Offset}},$$

where $V_{0}$ and $V_{\mathrm{Offset}}$ determine the amplitude and offset. $f_{0}$ and $f_{1}$ correspond to the base and envelop frequencies, respectively, while $f_{2}$ and Δf determine the time-dependent frequencies. $\phi_{1}-\phi_{3}$ are the initial phases. All of these can be parameters. $V_{H}$ has nine parameters in total; on the other hand, we used a manual gas exposure system, which will be explained later. As a result, it is still difficult to optimize all the parameters at the same time, even though BO is used. Then, taking into account that this study aims to prove the applicability of BO to $V_{H}$ optimization, we employed $V_{0}$ and $V_{\mathrm{Out}}$ as optimized parameters, whereas the rest of the parameters were fixed to the value employed in the previous study [34]. The $V_{H}$ waveform employed in this study, which has a 5 s duration, is shown in Figure 1. As described above, $V_{0}$ and $V_{\mathrm{Offset}}$ determine the amplitude and the offset of $V_{H}$, respectively, and hence, determine the voltage range. Although the response under the temperature modulation is influenced by the change rate in temperature and even hysteresis effects [33], the temperature range determined by $V_{0}$ and $V_{\mathrm{Offset}}$ is the most influential on the response. For this reason, we selected them as the parameters to be optimized.

### 2.2. Optimization Sequence

**Figure 2:**
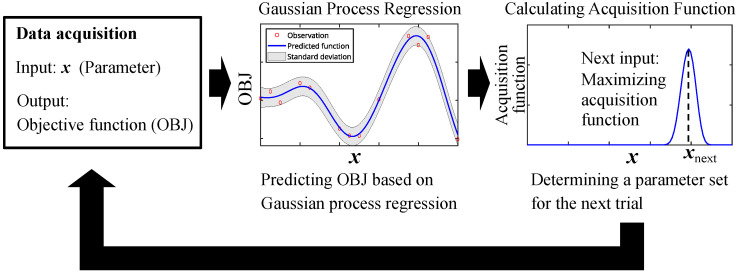
Schematic illustration of the concept of Bayesian optimization.

**Figure 3:**
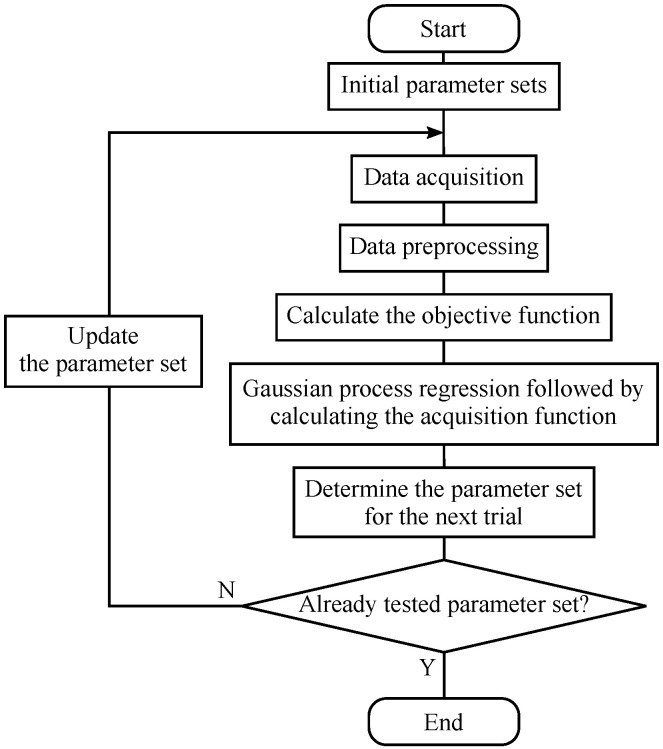
Schematic illustration of the optimization procedure based on Bayesian optimization.

As the schematic concept of BO is shown in Figure 2, BO is a method for finding inputs that maximize (or minimize) the output, the objective function (OBJ), based on GPR. From the observations, the OBJ, usually a black-box function, is predicted by GPR, based on which an acquisition function is calculated. Then, the parameter set that maximizes the acquisition function is chosen for the next trial. In this study, the optimization utilizing BO is conducted as follows, where the schematic sequence of the optimization is shown in Figure 3. First, sufficient data were acquired using $V_{H}$ with a certain parameter set, followed by preprocessing. Based on the preprocessed data, the OBJ was calculated. After that, the parameter set for the next trial was determined by the procedure described above. Data were again acquired after updating the parameter set. The optimization procedure was repeated until the parameter set for the next trial became the same one that had already been tested before. Although the optimization procedure shown in Figure 3 comprises the acquisition of calibration data and preprocessing including feature vector construction, which are also the parts of an e-nose, they may be engineered depending on the design concept of the e-nose. Namely, what kind of calibration data are acquired and/or how feature vectors are constructed should be appropriately decided considering the target of an e-nose, which is also an important topic. On the other hand, the main purpose of this study is the optimization of the temperature modulation. Hence, instead of setting a particular target, the conditions of data acquisition and preprocessing were employed to be typical ones at a laboratory, while referring to previous studies [33,34]. The details of data acquisition, analysis including preprocessing and classification, and BO conditions will be described in the following sections. All the analyses were carried out by MATLAB.

### 2.3. Data Acquisition Procedure

Sensor responses were measured during $V_{H}$ application under exposure to test gases. In this study, micro-electromechanical systems (MEMS)-based semiconductor gas sensors (TGS8100, Figaro Eng. Inc., Osaka, Japan) [44] were used. Unfortunately, they have been discontinued; however, the method we propose is applicable to all types of chemiresistor sensors as long as they can be operated with temperature modulation, taking into account the following. As described later, BO just uses the OBJ value, which may be calculated in any way from the sensor output; although, we employed frequency spectra of conductance followed by principal component analysis (PCA). In a similar manner, no limitation is imposed on sensor characteristics except for the capability of temperature modulation. Hence, the conclusion will hold for the other sensors.

**Figure 4:**
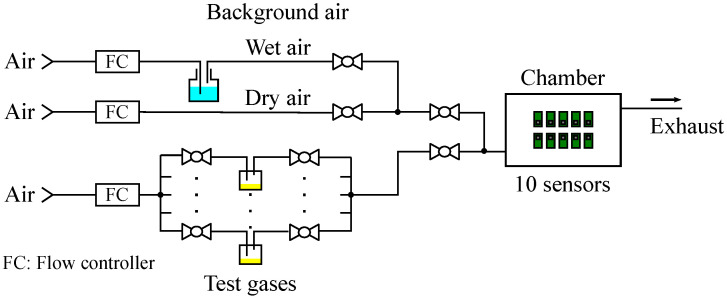
Schematic illustration of the flow-control system.

To expose the sensors to test gases, a custom-made flow-control system was used as schematically shown in Figure 4. The test gases were introduced into a test chamber as headspace gases, for which the liquid gas sources were evaporated. Aside from the test gas line, the system has a background (BKG) gas line to dilute the test gases, and it consists of a dry line and a wet line, allowing the control of chamber humidity. Synthesized air (N_2_ 79% + O_2_ 21%) was used for BKG gas as well as the carrier gas of the headspace gases. Five kinds of test gases, which were used as calibration gases for a commercial e-nose system (FF-2020, Shimadzu Corp., Kyoto, Japan), were used in this study, as shown in Table 1. The responses were obtained for five concentrations by changing the flow rate of the headspace gas, while the flow rate and relative humidity of BKG gas were maintained at 2 L/min and approximately 50%, respectively.

The concentrations were roughly estimated assuming that the partial pressure of test gases in the headspace gases reached their vapor pressures [45]. Although the estimated gas concentration ranges are different among the gases, we did not aim to quantify concentrations and thus did not try to equalize the concentrations. On the contrary, taking into consideration that the sensor response itself significantly differs between gases, the same concentration is not always an appropriate choice. Additionally, the responses having significantly different magnitudes may affect the classification rate, making classification problems easier, even though they are normalized. Instead, in this study, we tried to control the concentrations (or flow rates) so that the magnitudes of overall responses became similar among the gases to evaluate the classification rates in a fair manner. Note that the source of ammonia we used is an aqueous solution, and therefore, the concentration of ammonia in the solution could change during the measurements. However, it is difficult to accurately measure the gas concentration during the measurements at this stage. In that sense, we showed the gas concentration as a “rough estimate”. On the other hand, the obtained data were normalized to suppress the concentration information, aiming to classify the gas species. Taking normalization into account, the accurate control of gas concentration is not necessary, and therefore, the concentration inaccuracy may not affect the analysis results.

The ambient temperature in the chamber is also an important factor influencing the sensor response. Although we did not measure the ambient temperature in the chamber directly, we employed a flow-through system as shown in Figure 4, and therefore, it is reasonable to consider that the ambient temperature is almost the same as that of the inlet gas (approximately 24 °C). At least, the variation in the temperature among the measurements may be negligible, given that the flow rate of the BKG gas was kept constant (2 L/min).

**Figure 5:**
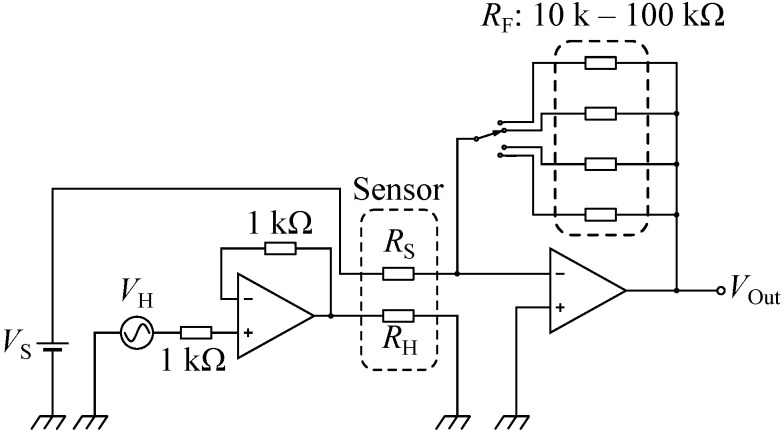
Schematic electrical circuit for the measurements. The current for heater resistor ($R_{H}$) was amplified by a voltage follower, while the sensor conductance ($G_{S}=1/R_{S}$) was converted to the output voltage ($V_{\mathrm{Out}}$) by an inverting amplifier.

Since the test chamber is allowed to install ten sensors, ten data were able to be obtained simultaneously. As shown in Figure 5, each sensor was connected to an electrical circuit, which supplied the heater current and output the sensor response. $V_{H}$ was input into a voltage follower, which amplified the current to be supplied to the heater resistor ($R_{H}$), while the voltage gain was almost in unity. We employed the sensor conductance ($G_{S}=1/R_{S}$) under a constant bias voltage ($V_{S}$) of 1.0 V as the response. $G_{S}$ was converted to the output voltage ($V_{\mathrm{Out}}$) by an inverting amplifier as follows:

(2) $$V_{\mathrm{Out}}=-G_{S}R_{F}V_{S},$$

where $R_{F}$ is a feedback resistor, which determines the voltage gain. We prepared four levels of $R_{F}$ in the range of 10kΩ–100kΩ taking account of the range of the sensor resistance (approx. 10kΩ–300kΩ). During the measurements, $V_{H}$ was generated synchronously, and $V_{\mathrm{Out}}$ was acquired at a sample rate of 10 kHz, where NI-9264 and NI-9201 (Emerson Electric Co., St. Louis, MO, USA) were used for the $V_{H}$ generation and the data acquisition, respectively. $R_{F}$ was appropriately chosen so that a good signal-to-noise ratio was achieved as much as possible. The electrical measurements were carried out by a program written by LabVIEW. As described above, five concentrations were employed for each of the gases, and ten duplicate measurements with ten sensors were carried out for each condition. As a result, 2500 data were acquired (5 gases × 5 concentrations × 10 measurements × 10 sensors) for each parameter set.

### 2.4. Analysis Procedures

#### 2.4.1. Data Preprocessing

$G_{S}$ was first calculated from the measured $V_{\mathrm{Out}}$ according to Equation (2) and then normalized to suppress the differences in the magnitude among the data originating from the gas concentration/sensitivity and sensor variation by the following equation:

(3) $$G_{S,n}=\frac{G_{S}-G_{S,\min}}{G_{S,\max}-G_{S,\min}}.$$

$G_{S,\max}$ and $G_{S,\min}$ indicate maximum and minimum conductance in the corresponding data. The frequency spectrum of $G_{S,n}$ was derived by fast Fourier transform (FFT). A data vector was built from the amplitude of the spectrum by taking the data points in an appropriate range at an interval of 0.2 Hz, the minimum frequency step. After applying the procedure for building a data vector to all the collected data, the data vectors were combined into a matrix. A dimensionality reduction was carried out by PCA. The first principal component (PC1) to PC4 was employed as a feature vector, and then, the OBJ for BO was calculated from the feature matrix as described later.

#### 2.4.2. Machine Learning Algorithm for Classification

ML-based gas classification was also conducted on the data for each of the parameter sets. The applicability of the proposed method to parameter optimization was investigated by evaluating the classification accuracy through optimization. For this purpose, the support vector machine (SVM) with a linear kernel, which is frequently used for gas classification [46,47], was employed to build a classification model. Although the linear SVM is common as a gas classification algorithm, nonlinear models like the k-nearest neighbor and the SVM with radial basis function (RBF) can output high accuracy compared with linear models, particularly in the case where a large amount of data are collected as in this study. On the other hand, the study aims to examine whether the optimization method is effective or not, that is, how the accuracy improved through the optimization. Hence, the models that always output high accuracy are not suitable for investigating the applicability of the optimization. Additionally, linear models usually show superior generalization performance to nonlinear models, which easily result in overfitting. Then, we employed the linear SVM to achieve a high classification accuracy through optimization even with a linear model. To apply the SVM, which is a binary learning algorithm, to multi-class classification, the error correcting output codes (ECOCs) algorithm was employed [48]. The details of the algorithm are described in Appendix A. The algorithm is provided as a MATLAB function [49].

The classification accuracy was evaluated based on 5-fold cross-validation with the following procedure. First, the data vectors (2500 data) were randomly separated into 5 partitions (500 each), where one partition was used as test data, while the rest of the data as training data. Then, PCA was carried out on the training data. Employing four-dimensional data from PC1 to PC4, a classification model was constructed. Then, test data were projected into the PC space by multiplying the coefficient matrix of the PCA obtained on the training data. The model accuracy for classification was evaluated using the test data. The procedure was repeated 5 times while changing the partition used as test data. Finally, average, maximum, and minimum accuracies were calculated.

## 3. Bayesian Optimization Conditions

### 3.1. Objective Function

As BO is a method to maximize (or minimize) an OBJ, usually a black-box function, the choice of an OBJ has a decisive effect on the optimization result. For gas classification, classification accuracy is seemingly the most suitable and straightforward quantity as an OBJ. However, the accuracy greatly depends on ML algorithms; particularly, high accuracy is easily obtained when a nonlinear classification algorithm and high-dimensional feature vectors are used to build the classification model. On the other hand, such a model can fall into overfitting and lose generalization. Additionally, complex models such as one based on neural networks need large training costs. Taking account of these concerns, we eschewed the use of accuracy as an OBJ. Instead, the Bouldin–Davies index (DBI) [50] was employed. In this study, the similarity measure between clusters (test gases in this study) *i* and *j* is defined as

(4) $$D_{ij}=\frac{\sigma_{i}+\sigma_{j}}{d(c_{i},c_{j})}.$$

$\sigma_{i}$ is the mean distance of all elements from the centroid in the cluster $i,\;c_{i}$, while $d(c_{i},c_{j})$ is the distance between the centroids of cluster *i* and *j*. With $D_{ij}$, DBI is then defined as

(5) $$DBI=\frac{1}{n}\sum_{i=1}^{n}\max_{i\neq j}D_{ij},$$

where *n* is the number of clusters. Equation (4) indicates that $D_{ij}$ becomes smaller as the variation within a cluster decreases and the distance between the clusters increases. Given averaging the maximum $D_{ij}$ with respect to the clusters, the DBI can be a good measure of the separation of clusters [51]. The DBI was calculated using the PC scores from PC1 to PC4, and the subsequent optimization procedure aimed to minimize the DBI. PCA for calculating the DBI was conducted using all the data for each of the parameter sets.

### 3.2. GPR and Acquisition Function

After calculating the DBI, a GPR model was built using the DBI and the parameter values ($V_{0}$ and $V_{\mathrm{Offset}}$). Then, an acquisition function was calculated to determine the next parameter set, where expected improvement [40] was employed as an acquisition function. The parameter set that maximizes the acquisition function was chosen for the next trial. The range of the parameter set was restricted to $0.5\;V\;\leq \;2V_{0}+V_{\mathrm{Offset}}\leq \;1.8\;V$ with a 0.05 V step. The upper limit was decided according to the sensor ratings ($V_{H}=1.8$ V), while the lower bound was decided to be the value above which a significant response was obtained according to the measurement data. Under the constraint, the number of candidates was approximately 500.

## 4. Results and Discussion

### 4.1. Sensor Response and Data Preprocessing

**Figure 6:**
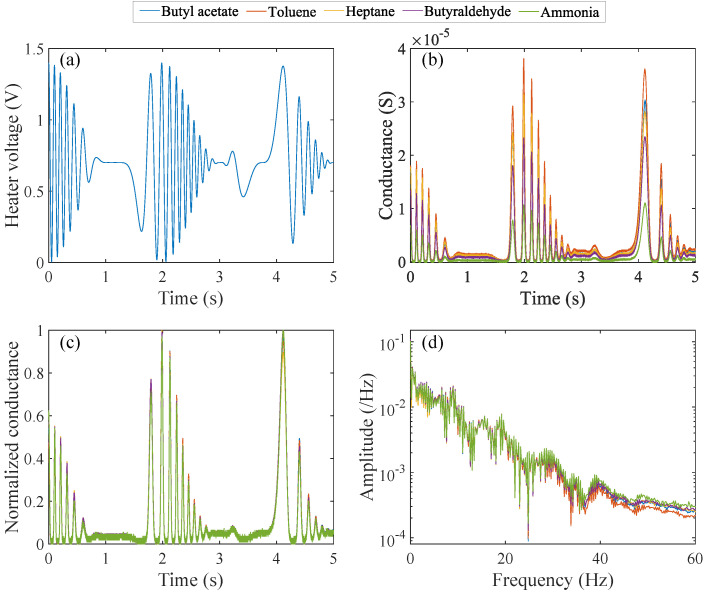
(**a**) Heater voltage with one of the initial parameter sets ($V_{0}$: 0.35 V, $V_{\mathrm{Offset}}$: 0.75 V) and one of the corresponding measurement results for each of the test gases: (**b**) $G_{S}$, (**c**) $G_{S,n}$, and (**d**) frequency spectra of $G_{S,n}$.

Figure 6b–d shows one of the measurement and preprocessing results under the application of Figure 6a $V_{H}$, where 0.35 and 0.70 V were employed for $V_{0}$ and $V_{\mathrm{Offset}}$, respectively. The results for the flow rate of 0.5 sccm for all the gases in the fifth cycle are displayed. $G_{S}$ (Figure 6b) varies in response to $V_{H}$ due to the change in the sensor temperature according to the Arrhenius law:

(6) $$G_{S}=G_{0}\exp\left(-\frac{E_{a}}{k_{B}T}\right),$$

where $E_{a}$, $k_{B}$, *T* denote the activation energy of conductance, Boltzmann constant, and temperature, respectively. Under gas exposure, the factor $G_{0}$ changes according to gas species and concentrations. Additionally, their influences are modulated by the operating temperature and even its hysteresis [32,33]. As a result, the different $G_{S}$ depending on the gases as shown in Figure 6b. Then, $G_{S,n}$ was derived according to Equation (3) to suppress the differences in the magnitude of $G_{S}$ (Figure 6c). The frequency spectra of $G_{S,n}$ calculated by FFT are shown in Figure 6d, where the vertical axis is displayed in a logarithmic scale. The spectra have non-negligible amplitude from 0 Hz to about 35 Hz, and hence, the data points were collected in the frequency range of 0.2–35 Hz to build a data vector with 175 dimensions for each of the measurement results. Then, the data vectors were combined into a matrix with a dimension of 2500×175. PCA was carried out on the data matrix to obtain the feature matrix as described in Section 2.4.1.

**Figure 7:**
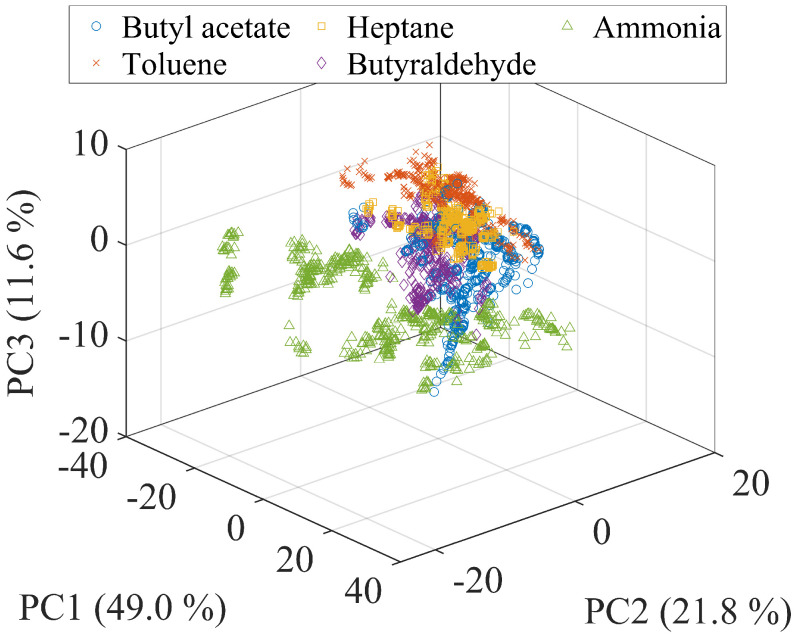
PC plot of the data obtained using the heater voltage shown in Figure 6a.

The results of PCA conducted on the dataset for $V_{H}$ shown in Figure 6a is plotted in Figure 7. The distribution of most of the gas species are overlapped with each other; although, the distribution of ammonia was relatively well separated. The mean classification accuracy on the dataset was 85.4%, which is not high enough compared with that in the previous study [33], and hence, we confirmed that it is necessary to find an optimal parameter set.

### 4.2. Minimizing Objective Function

**Figure 8:**
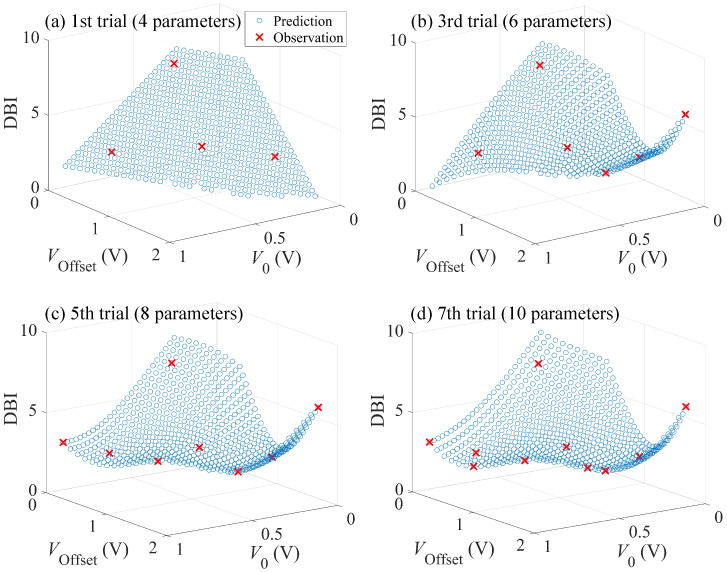
GPR results after (**a**) first, (**b**) third, (**c**), fifth, and (**d**) seventh trials. The blue circles indicate the predicted mean obtained by the regression, while the red crosses the experimental results.

**Figure 9:**
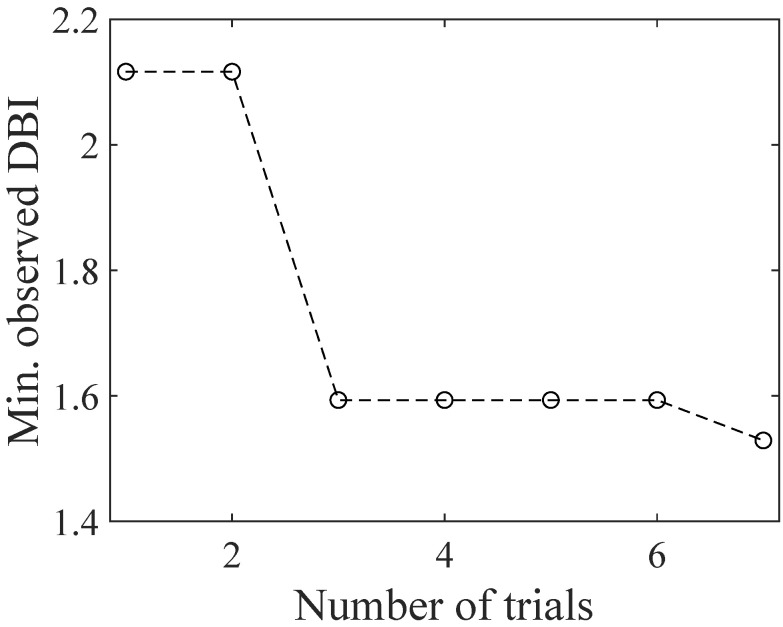
The observed minimum DBI plotted as a function of the number of trials.

Figure 8a–d show the results of GPR after several trials, where the z-axis shows the DBI plotted as a function of $V_{0}$ and $V_{\mathrm{Offset}}$. Blue circles and red crosses, respectively, represent the predicted mean and the observations of DBI. At the first trial, four parameter sets, which were properly chosen to distribute across the entire range of the parameter space, were tested. As the number of observations increased, the regression result revealed the existence of two minima, which approximately correspond to the parameter sets: ($V_{0}$, $V_{\mathrm{Offset}}$) = (0.3 V, 1.15 V) and (0.75 V, 0.3 V). The observation showed that the latter set resulted in a smaller DBI. The GPR predicted mean indicates that the DBI tends to become smaller as $2V_{0}+V_{\mathrm{Offset}}$ becomes larger. After seven trials (ten parameter sets tested), the predicted next (eighth) parameter set was the same as the previous (seventh) one, and then, the trial was stopped. As a result, the DBI was decreased from 2.1 to 1.5 through seven trials as shown in Figure 9, where the minimum DBI observed through the trials is plotted as a function of the number of trials. The result demonstrates the successful reduction in the DBI based on BO.

### 4.3. Gas Classification and Validation of the Proposed Method

Although it was demonstrated in the previous section that the DBI, the OBJ, was minimized by BO, the link between the DBI and the classification accuracy is not shown yet. Then, in this section, the validity of BO for optimizing the parameters in temperature modulation was discussed in terms of the ML-based gas classification.

**Figure 10:**
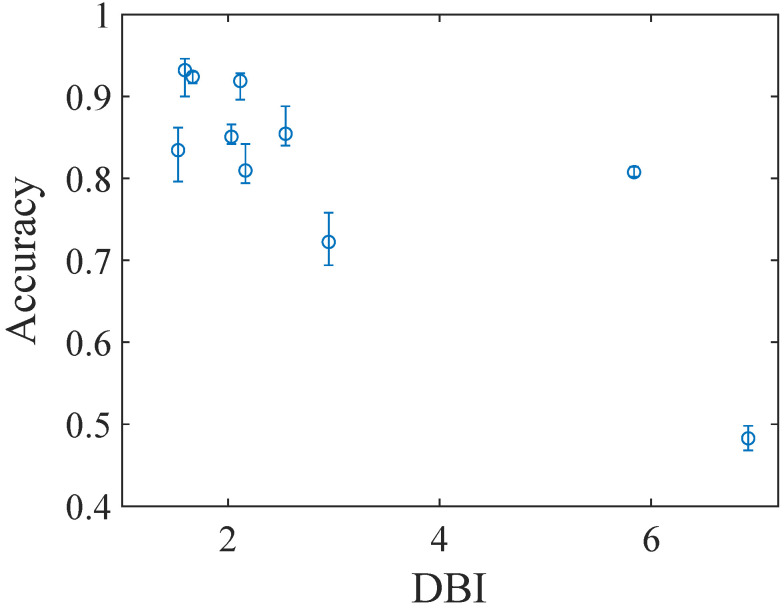
Classification accuracy plotted as a function of the DBI. The markers and the error bars indicate mean and max./min. accuracies, respectively.

Figure 10 shows the accuracy of gas classification plotted as a function of the DBI. The markers and error bars indicate the mean and maximum/minimum values, respectively. The overall accuracy increases with the decrease in the DBI, which indicates that the DBI acts as a good OBJ. The accuracy itself also improved from 85.4%, obtained for the parameter set first tested, to 93.2% for the optimal parameter set. These results demonstrate the validity of the proposed optimization method. Although it was indicated that the DBI can be a good OBJ for optimization, parameter sets that exhibited relatively high and low accuracy were observed for a comparable DBI in the range of less than 2.5. The parameter sets that showed high and low accuracy correspond to the two minima observed in the GPR regression result shown in Figure 8.

In order to gain an insight into the link between the accuracy and the DBI, the elements of the similarity measure $D_{ij}$ were examined, and a clear difference in the variance of $D_{ij}$ was found:

(7) $$\mathrm{Var}\left[D_{ij}\right]\;\mathrm{for}\;i>j.$$

Table 2 summarizes the classification accuracy, the DBI, and the variance in $D_{ij}$ associated with the values of the parameters $V_{0}$ and $V_{\mathrm{Out}}$. The rows are aligned in order of increasing DBI. It is observed that higher accuracies were obtained for the parameter sets exhibiting the larger variance in $D_{ij}$ when the DBI are comparable with each other, e.g., the parameter sets of #1, 2, and 3. With these three parameter sets, a clear difference in the accuracy was observed: 92–93% for #2 and 3, whereas it was 83% for #1, despite the similar DBI in the range of 1.5–1.7. In contrast, the variances in $D_{ij}$ are 0.090, 0.211, and 0.276 for #1, 2, and 3, respectively, demonstrating the parameter sets that exhibited higher variances (#2 and 3) exhibited higher accuracies than those with smaller variances (#1). The tendency was similar for parameter sets #4, 5, and 6, which exhibited the DBI in the range of 2.0–2.2. Although the accuracy does not increase monotonically with increasing the variance in $D_{ij}$ for the similar DBI, the tendency indicates that the variance in $D_{ij}$ may be also a good measure of the separability of clusters, when the obtained DBIs are similar to each other.

As described in Equation (A1), the label of the test data is determined so that the total loss calculated on each of the learners, which was constructed for all pairs, is minimized. Accordingly, the accuracy, evaluated with the labels, depends on the separability between clusters of all pairs. It is expected that a pair of least separable clusters results in a comparable loss for each class and thus affects the accuracy most. Given that the DBI is calculated by averaging the largest $D_{ij}$, namely, the least separable clusters, with respect to each cluster, *i*, then, it is reasonable to consider the DBI as a measure of separation of the clusters. On the other hand, when the parameter sets exhibit comparable DBI with each other, not only the least separable pair but also the other pairs are influential in causing the difference in the accuracy. The large variance indicates that there are more $D_{ij}$ with relatively small values. A small $D_{ij}$ indicates a good separability between the clusters *i* and *j*. Since a coding matrix by all pairs was employed in this study, it is expected that the model outputs become more accurate as more cluster pairs have small $D_{ij}$. This leads to a larger variance in $D_{ij}$ under the assumption of similar DBI among the parameter sets. Therefore, both the DBI and the variance in $D_{ij}$ should be taken into account to further refine the OBJ for optimization, which would be addressed in future work.

## 5. Conclusions

In order to facilitate the determination process of the heater waveform in the temperature modulation with a reduced number of calibration tests, BO was employed. Its applicability was investigated by optimizing the parameters, which determine the range of heater voltage. The optimization was carried out to minimize the DBI, which was employed as the OBJ. After seven trials with 10 parameter sets tested, the OBJ was successfully reduced from 2.1 to 1.5. Additionally, the classification accuracy roughly increased with decreasing the DBI, indicating that the DBI is a suitable OBJ. On the other hand, it was also revealed that there is still room for improvement in the OBJ in terms of incorporating the variance of the similarity measure. After the optimization process, the accuracy increased from 85.4% to 93.2%, demonstrating that BO is promising for the optimization of temperature modulation. Furthermore, the method we introduced defined only the OBJ, which is easily calculated from the feature vectors and generally applied to gas classification problems. As a consequence, this study paves the way for BO-based optimization as a general method for the temperature modulation. Then, as a next step toward a compact and low-cost e-nose with high classification accuracy, model construction must be addressed, which requires comprehensive investigation including data acquisition, feature vector construction, and engineering a classification algorithm. Besides the accuracy, the robustness of a model is also an important factor, which has been mainly addressed in terms of post-processing; on the other hand, it is known that the quality of calibration data also affects the robustness [52]. However, studies on the robustness are still limited concerning the data under temperature modulation, in particular, using such a complex heater waveform as in this study. Hence, in addition to the improvement in the objective function as discussed above, comprehensive studies are necessary for future work.

## Figures and Tables

**Table 1:** Gas species employed in this study. The flow rates for headspace gas and the estimate of corresponding concentrations are also displayed.

Gas Species	Source Purity (wt%)	Flow Rates of Headspace Gas (sccm)	Concentration Estimate (ppm)
Butyl acetate	99.0	0.5, 1.0, 1.5, 2.0, 2.5	4–19
Toluene	99.8	0.5, 1.0, 1.5, 2.0, 2.5	9–47
Heptane	99.0	0.5, 1.0, 1.5, 2.0, 2.5	15–76
Butyraldehyde	98.0	0.5, 0.9, 1.3, 1.6, 2.0	37–150
Ammonia (aq.)	28.0–30.0 *	0.5, 1.0, 1.5, 2.0, 2.5	37–180

* The concentration of ammonia.

**Table 2:** The DBI, the variance of $D_{ij}$, and the mean accuracy for each of the parameter sets.

Label	$V_{0}$ (V)	$V_{\mathrm{Offset}}$ (V)	DBI	Var [$D_{\mathrm{ij}}$]	Accuracy (Mean)
#1	0.75	0.3	1.5	0.090	0.834
#2	0.3	1.2	1.6	0.211	0.932
#3	0.35	1.05	1.7	0.276	0.924
#4	0.575	0.65	2.0	0.208	0.850
#5	0.1	1.2	2.1	0.794	0.919
#6	0.7	0.2	2.2	0.323	0.810
#7	0.35	0.7	2.5	0.269	0.854
#8	0.9	0	3.0	1.776	0.722
#9	0.025	1.75	5.8	11.33	0.808
#10	0.3	0.1	6.9	7.382	0.483

## Data Availability

The raw data supporting the conclusions of this article will be made available by the authors on request.

## Appendix A. ECOC Algorithm

In the algorithm of ECOC, a cording matrix $M\in {\left\{-1,0,1\right\}}^{k\times l}$ based on all-pairs design, where *k* and *l* indicate the number of classes and binary learners (here, l=k(k−1)/2), respectively, was used. Prediction was carried out using loss-based decoding according to the following equation:

(A1) $$\hat{y}=\min_{i}\frac{\sum_{j=1}^{l}\left|m_{ij}\right|L\left(m_{ij},f_{j}\left(x\right)\right)}{\sum_{j=1}^{l}\left|m_{ij}\right|},$$

where $\hat{y}$ is the predicted class for the input x (here the scores of PC), $m_{ij}$ is the element (i,j) of M, and $f_{j}\left(x\right)=w\cdot x+b$ is the predictor on *j*-th learner. The loss function L(s,t) is the “hinge” function expressed as $L\left(s,t\right)=\max\left[0,1-st\right]/2$.
